# Safety and efficacy of vismodegib in patients with basal cell carcinoma nevus syndrome: pooled analysis of two trials

**DOI:** 10.1186/s13023-016-0506-z

**Published:** 2016-09-01

**Authors:** Anne Lynn S. Chang, Sarah T. Arron, Michael R. Migden, James A. Solomon, Simon Yoo, Bann-Mo Day, Edward F. McKenna, Aleksandar Sekulic

**Affiliations:** 1 Stanford University, 450 Broadway, Pavilion C, 2nd floor, Redwood City, CA 94063 USA; 2 University of California, San Francisco, 1701 Divisadero Street, Box 0316, San Francisco, CA 94115 USA; 3 The University of Texas MD Anderson Cancer Center, Unit 1452, 1400 Pressler Street, Houston, TX 77030 USA; 4 Ameriderm Research, 725 West Granada Boulevard, Suite 44, Ormond Beach, FL 32174 USA; 5 University of Central Florida, CI6850 Lake Nona Boulevard, Orlando, FL 32827 USA; 6 University of Illinois, 506 South Mathews Avenue, Urbana, IL 61801 USA; 7 Northwestern University, 676 North St. Clair Street, Suite 1600, Chicago, IL 60611 USA; 8 Genentech, Inc., 1 DNA Way, South San Francisco, CA 94080 USA; 9 Mayo Clinic, 13400 East Shea Boulevard, Scottsdale, AZ 85259 USA

**Keywords:** Basal cell carcinoma, Vismodegib, Basal cell carcinoma nevus syndrome

## Abstract

**Background:**

Aberrant activation of the Hedgehog (Hh) pathway is a key driver in the pathogenesis of basal cell carcinomas (BCCs), including patients with BCC nevus syndrome (BCCNS). It is unclear whether BCCs arising in patients with BCCNS respond differently to vismodegib than in patients without BCCNS. We examined the best overall response rate (BORR) and adverse events (AEs) of vismodegib in patients with advanced BCC (aBCC) with and without BCCNS.

**Methods:**

Patients were treated with vismodegib 150 mg/day in the ERIVANCE BCC trial (ClinicalTrials.gov number, NCT00833417) and the expanded access study (EAS; ClinicalTrials.gov number, NCT01160250). BCCNS diagnosis was based on medical history at the time of enrollment. Metastatic BCC response was evaluated using Response Evaluation Criteria In Solid Tumors, version 1.0 (RECIST v1.0) in both studies. Locally advanced BCC was evaluated by a novel composite end point in ERIVANCE BCC and by RECIST v1.0 in the EAS. Response assessments were performed every 8 weeks in ERIVANCE BCC and every 8–16 weeks in the EAS. Safety assessments (National Cancer Institute Common Terminology Criteria for Adverse Events, version 3.0) were performed monthly in both trials. Because of described differences in response assessment/schedule, patients with BCCNS were not pooled across trials. Analytic cohorts for BCCNS and sporadic aBCC were created within each trial for comparison using descriptive statistical methods.

**Results:**

Forty-one patients with BCCNS were included in the study: 22 from ERIVANCE BCC and 19 from the EAS. Investigator-assessed BORR in BCCNS groups ranged from 31 to 81 % in patients with locally advanced BCC (*n* = 33) and was 50 % in patients with metastatic BCC (*n* = 6). These results were comparable with the non-BCCNS groups. Incidence and severity of AEs were also comparable between the BCCNS and non-BCCNS groups. Amenorrhea was observed in both patient cohorts and was reversible in two patients who discontinued treatment.

**Conclusion:**

Vismodegib demonstrated comparable efficacy and safety against aBCC in patients with and without BCCNS.

## Background

First described in patients with basal cell carcinoma (BCC) nevus syndrome (BCCNS), aberrant activation of the Hedgehog (Hh) pathway is a key pathogenic driver in BCC [1, 2]. The majority of genetic alterations in the Hh pathway are loss-of-function mutations in the tumor-suppressor gene *PTCH1* [2, 3]. Patients with BCCNS develop dozens of BCCs over their lifetimes [4], including unresectable advanced BCCs (aBCCs) that are either locally advanced (laBCC) or metastatic (mBCC).

Therapeutic options are limited for patients with aBCC. Vismodegib, the first Hh pathway inhibitor (HPI) approved by the US Food and Drug Administration (FDA), is indicated for patients who have aBCC that has recurred after surgery or who are not candidates for surgery and radiation [5]. In its pivotal approval study (ERIVANCE BCC), vismodegib demonstrated an overall response rate (ORR) of 43 % in patients with laBCC and 30 % in patients with mBCC by independent review [5]. In addition, vismodegib reduced the size of existing BCC lesions and prevented development of new lesions compared with placebo in 41 patients with BCCNS with multiple surgically eligible BCCs who were enrolled in an investigator-sponsored trial [6].

It is unclear whether BCCs arising in patients with BCCNS respond differently to vismodegib than in patients without BCCNS. Here, we investigate the efficacy and safety of vismodegib in patients with aBCC with and without BCCNS enrolled in the ERIVANCE BCC pivotal trial [5] and the US expanded access study (EAS) [7].

## Methods

### Study design and treatment

This was a pooled analysis of two similar open-label clinical trials. ERIVANCE BCC (ClinicalTrials.gov number, NCT00833417) was an international, multicenter, noncomparative phase 2 study. EAS (ClinicalTrials.gov number, NCT01160250) was a multicenter, open-label, noncomparative expanded access study to provide patients with aBCC access to vismodegib prior to regulatory approval, and was terminated early due to FDA approval. Patients received oral vismodegib 150 mg/day until disease progression, intolerable toxicity, patient withdrawal, or study termination. All patients signed written informed consent.

### Key eligibility criteria

Key eligibility criteria for the ERIVANCE BCC and EAS studies were similar. Patients with mBCC had histologic confirmation of distant metastasis. Patients with laBCC had ≥1 lesion measuring ≥10 mm, inoperable or surgery contraindicated, and prior radiation to ≥1 lesion, unless contraindicated or inappropriate. Other criteria included age ≥18 years, adequate organ function, and Eastern Cooperative Oncology Group performance status ≤2. Both trials used Response Evaluation Criteria In Solid Tumors, version 1.0 (RECIST v1.0) for assessment of mBCC and allowed enrollment of patients with BCCNS as long as all other eligibility criteria were met. The EAS also used RECIST v1.0 for assessment of patients with laBCC. ERIVANCE BCC defined response as a ≥30 % decrease in the externally visible or radiographic dimension (if applicable) or complete resolution of ulceration (if present at baseline). BCCNS diagnosis was based on medical history at the time of enrollment and/or assessment of clinical investigator.

### Assessments

#### Efficacy assessments

Response assessments were performed every 8 weeks in ERIVANCE BCC and every 8–16 weeks in the EAS.

#### Safety assessments

Adverse events (AEs) were assessed on a monthly basis in both trials and graded according to National Cancer Institute Common Terminology Criteria for Adverse events, version 3.0.

### Analysis

All patient data available as of November 26, 2010 for ERIVANCE BCC (primary analysis) and April 23, 2012 for US EAS (final analysis) were included in the analyses. Analytic cohorts for BCCNS and non-BCCNS were created within each trial for comparison using descriptive statistical methods.

Data were not pooled across the trials because of the described differences in the schedule and the criteria for assessment of response. Best ORR (BORR) was analyzed in efficacy-evaluable patients. Clopper-Pearson 95 % confidence intervals (CIs) were computed.

## Results

### Patient characteristics

The ERIVANCE BCC trial enrolled 104 patients: 71 (68 %) with laBCC and 33 (32 %) with mBCC. Twenty-two (31 %) patients with BCCNS had laBCC; no patients with BCCNS had mBCC (Table 1). The EAS study enrolled 119 patients: 62 (52 %) with laBCC and 57 (48 %) with mBCC. Twelve (17 %) patients with BCCNS had laBCC; 7 (12 %) patients with BCCNS had mBCC. Baseline demographic and disease characteristics were generally comparable between patients with and without BCCNS, except for younger age and higher number of women of childbearing potential (WCBP) in the BCCNS cohort.

**Table 1:** Patient demographics and baseline disease characteristics^a^

	Erivance BCC (*N* = 104)			EAS (*N* = 119)			
	laBCC		mBCC	laBCC		mBCC	
	BCCNS (*n* = 22)	Non-BCCNS (*n* = 49)	Non-BCCNS (*n* = 33)	BCCNS (*n* = 12)	Non-BCCNS (*n* = 50)	BCCNS (*n* = 7)	Non-BCCNS (*n* = 50)
Median age, years (range)	47 (21–71)	67 (38–101)	62 (38–92)	52 (26–79)	67 (40–92)	58 (37–71)	63 (24–100)
Female, *n* (%)	10 (45)	22 (45)	9 (27)	6 (50)	13 (26)	3 (43)	9 (18)
WCBP, *n* (%)	3 (33)	1 (2)	2 (6)	4 (33)	2 (4)	1 (14)	1 (2)
ECOG PS, *n* (%)							
0–1	22 (100)	44 (90)	32 (97)	12 (100)	46 (92)	7 (100)	45 (90)
2	0	5 (10)	1 (3)	0	4 (8)	0	5 (10)
Target lesions, *n* (%)							
1	13 (59)	35 (71)	9 (27)	4 (33)	30 (60)	4 (57)	20 (40)
2	4 (18)	8 (16)	4 (12)	2 (17)	11 (22)	0	10 (20)
≥3	5 (23)	6 (12)	20 (61)	6 (50)	9 (18)	3 (43)	20 (40)
Prior treatment, *n* (%)							
Surgery	21 (96)	41 (84)	32 (97)	12 (100)	45 (90)	7 (100)	47 (94)
Radiotherapy	1 (5)	21 (43)	19 (58)	1 (8)	19 (38)	2 (29)	33 (66)
Systemic therapy	5 (23)	3 (6)	10 (30)	2 (17)	9 (18)	2 (29)	18 (36)
Surgery contraindicated, *n* (%)	18 (82)	25 (51)	NA	7 (58)	28 (56)	NA	NA

*BCCNS* basal cell carcinoma nevus syndrome, *EAS* expanded access study, *ECOG PS* Eastern Cooperative Oncology Group performance status, *laBCC* locally advanced basal cell carcinoma, *mBCC* metastatic basal cell carcinoma, *NA* not available, *WCBP* women of childbearing potential

^a^There were no patients with BCCNS with mBCC in the ERIVANCE BCC study; therefore, this column is omitted in the table

### Treatment exposure

Median duration of treatment with vismodegib was shorter in the EAS study (5.0–7.1 months across cohorts) than in the ERIVANCE BCC study (9.6–10.5 months across cohorts). Within each trial, the median treatment duration in patients with BCCNS was similar to the duration in patients without BCCNS. Median dose intensity was similar (>97 %) across all cohorts in both studies.

### Best overall response rate

Similar clinical activity was observed across all cohorts in both studies. In the ERIVANCE BCC study, the investigator-assessed BORR in patients with BCCNS with laBCC was 81 % (95 % CI: 58–95 %); in those without BCCNS, the BORR was 50 % (95 % CI: 34–66 %). Although this study suggested that patients with BCCNS with laBCC might be more responsive to vismodegib than patients without BCCNS, this pattern was not observed in the EAS, in which the BORR was 33 % (95 % CI: 10–65 %) in patients with BCCNS and 50 % (95 % CI: 35–65 %) in patients without BCCNS (Table 2).

**Table 2:** Investigator-assessed BORR (efficacy-evaluable patients) comparing BCCNS and non-BCCNS patient groups

	Erivance BCC (*N* = 96)			EAS (*N* = 95)			
	laBCC		mBCC	laBCC		mBCC	
	BCCNS (*n* = 21)	Non-BCCNS (*n* = 42)	Non-BCCNS (*n* = 33)	BCCNS (*n* = 12)	Non-BCCNS (*n* = 44)	BCCNS (*n* = 6)	Non-BCCNS (*n* = 33)
BORR, *n* (%) [95 % CI]	17 (81) [58–95]	21 (50) [34–66]	15 (46) [28–64]	4 (33) [10–65]	22 (50) [35–65]	3 (50) [12–88]	9 (27) [13–46]
Complete response	8 (38)	12 (29)	0	1 (8)	5 (11)	2 (33)	0
Partial response	9 (43)	9 (21)	15 (46)	3 (25)	17 (39)	1 (17)	9 (27)
Stable disease	3 (14)	12 (29)	15 (46)	6 (50)	21 (48)	3 (50)	17 (52)
Progressive disease	1 (5)	5 (12)	2 (6)	2 (17)	0	0	3 (9)
Not evaluable or missing	0	4 (10)	1 (3)	2 (17)	1 (2)	0	4 (12)

*BCCNS* basal cell carcinoma nevus syndrome, *BORR* best overall response rate, *CI* confidence interval, *EAS* expanded access study, *laBCC* locally advanced basal cell carcinoma, *mBCC* metastatic basal cell carcinoma

Among patients with mBCC, the BORR was 46 % (95 % CI: 28–64 %) in patients without BCCNS in the ERIVANCE BCC study; no mBCC was noted in patients with BCCNS. In the EAS, patients with BCCNS with mBCC had a BORR of 50 % (95 % CI: 12–88 %) and those without BCCNS had a BORR of 27 % (95 % CI: 13–46 %).

### Safety

No consistent trends in the incidence of AEs were observed across studies. The most frequent AEs in patients with BCCNS were alopecia (86 and 58 % in ERIVANCE BCC and the EAS, respectively), muscle spasms (77 and 63 %), weight decrease (68 and 5 %), and dysgeusia (59 and 74 %) (Table 3). The most frequent AEs in patients with non-BCCNS aBCC were alopecia (57 and 58 % in ERIVANCE BCC and the EAS, respectively), muscle spasms (66 and 72 %), weight decrease (40 and 18 %), and dysgeusia (49 and 70 %) (Table 3). The longer period of follow-up for ERIVANCE BCC compared with the EAS likely accounts for differences in later-onset AEs such as weight decrease. In both studies, there were lower percentages of dysgeusia in patients with BCCNS compared with patients without BCCNS. Incidences of grade 3–5 AEs were 41 % in the BCCNS group versus 43 % in the non-BCCNS group in ERIVANCE BCC, and 16 % versus 32 % in the EAS.

**Table 3:** Most common adverse events stratified by BCCNS or non-BCCNS status

Selected AEs, *n* (%)	Erivance BCC (*N* = 104)		EAS (*N* = 119)	
	BCCNS (*n* = 22)	Non-BCCNS (*n* = 82)	BCCNS (*n* = 19)	Non-BCCNS (*n* = 100)
Any AE	22 (100)	82 (100)	18 (95)	98 (98)
Grade 3–5 AE	9 (41)	35 (43)	3 (16)	32 (32)
Alopecia	19 (86)	47 (57)	11 (58)	58 (58)
Muscle spasms	17 (77)	54 (66)	12 (63)	72 (72)
Weight decreased	15 (68)	33 (40)	1 (5)	18 (18)
Dysgeusia	13 (59)	40 (49)	14 (74)	70 (70)
Nausea	9 (41)	21 (26)	3 (16)	20 (20)
Fatigue	8 (36)	29 (35)	7 (37)	16 (16)
Diarrhea	7 (32)	16 (20)	3 (16)	27 (27)
Arthralgia	5 (23)	11 (13)	2 (11)	2 (2)

*AE* adverse event, *BCCNS* basal cell carcinoma nevus syndrome, *EAS* expanded access study

For WCBP, amenorrhea or irregular menstruation was reported in 2/3 (67 %) and 0/3 patients with and without BCCNS, respectively, in the ERIVANCE BCC study. It was reported in 2/5 (40 %) and 2/3 (67 %) patients with and without BCCNS, respectively, in the EAS.

## Discussion

Vismodegib demonstrated clinical activity across all cohorts, including patients with BCCNS, confirming the efficacy of HPIs in these patients [2, 3]. While numerical differences in BORR were observed across cohorts, they were not consistent trends that were clinically significant. Rather, differences were likely due to similar but nonidentical response criteria, treatment duration, and length of follow-up in each study, as well as biologic factors that may affect treatment response (e.g., tumoral heterogeneity, presence of SUFU mutation).

Numerical differences were also observed between cohorts with respect to the incidence of various AEs; however, no consistent patterns were observed across the two studies. As patients with BCCNS tend to be younger than those without BCCNS, BCCNS cohorts also tend to include a higher proportion of WCBP than non-BCCNS cohorts. A survey of six phase 1 and 2 Roche-sponsored studies of vismodegib that included WCBP determined that irregular menses or amenorrhea occurred in 10 (28.5 %) of 35 premenopausal women (inclusive of the women presented in this analysis), eight of whom had BCCNS [8]. An analysis by BCCNS status was not performed. Results of hormonal evaluation were available for four patients after the onset of irregular menses or amenorrhea; two had normal values (premenopausal), one patient was considered postmenopausal, and one had elevated follicle-stimulating hormone, elevated luteinizing hormone, and normal estradiol values [8]. Menses resumed in two patients who discontinued vismodegib, suggesting that amenorrhea observed with vismodegib treatment might be reversible.

When stratified for BCCNS status in this analysis, amenorrhea was observed in WCBP in both groups. The mechanism responsible has yet to be fully elucidated, although the Hh pathway is known to play a role in follicular development and patients with BCCNS frequently develop ovarian cysts (25–50 % of patients) [4, 9]. Additionally, a recent case report suggested that vismodegib might induce amenorrhea by blockading follicle-stimulating hormone receptor-dependent signal transduction [10].

## Conclusions

Vismodegib demonstrates clinical activity in patients with aBCC with and without BCCNS. Overall, the safety profile was similar in both groups, an important consideration when counseling patients.

## Abbreviations

aBCC: Advanced basal cell carcinoma
AE: Adverse event
BCC: Basal cell carcinoma
BCCNS: Basal cell carcinoma nevus syndrome
BORR: Best overall response rate
CI: Confidence interval
EAS: Expanded access study
FDA: US Food and Drug Administration
Hh: Hedgehog
HPI: Hedghog pathway inhibitor
laBCC: Locally advanced basal cell carcinoma
mBCC: Metastatic basal cell carcinoma
ORR: Objective response rate
RECIST: Response evaluation criteria in solid tumors
WCBP: Women of childbearing potential
